# Recruiting students to rural longitudinal integrated clerkships: a qualitative study of medical educationists’ experiences across continents

**DOI:** 10.1186/s12909-023-04949-z

**Published:** 2023-12-19

**Authors:** Janani Pinidiyapathirage, Robert Heffernan, Brendan Carrigan, Sherrilyn Walters, Lara Fuller, Kay Brumpton

**Affiliations:** 1 Rural Medical Education Australia, 190 Hume Street, Toowoomba, QLD 4350 Australia; 2 School of Medicine and Dentistry, Griffith University, Gold Coast Campus, Southport, Australia; 3 Rural Community Clinical School, School of Medicine, Deakin University, Geelong, Australia

**Keywords:** Longitudinal integrated clerkships, Rural placements, Personally Arranged Learning Session (PeArLS)

## Abstract

**Introduction:**

Many health systems struggle in the provision of a sustainable and an efficient rural health workforce. There is evidence to suggest that Longitudinal Integrated Clerkships (LIC) placing student learners in rural community settings have positively impacted the provision of rural health care services The recruitment and engagement of students in rural LIC have significant challenges. This study explored best practice methods of recruiting and supporting the transition of medical students into rural LIC.

**Methods:**

The study took place during the 2021 Consortium of Longitudinal Integrated Clerkships Conference, a virtual event hosted by Stellenbosch University, South Africa. Participants consisted of delegates attending the Personally Arranged Learning Session (PeArLS) themed ‘Secrets to success’. The session was recorded with the participants’ consent and the recordings were transcribed verbatim. Data was uploaded to NVivo software and coded and analyzed using constant comparative analysis. Salient themes and patterns were identified.

**Results:**

Thirteen attendees participated in the PeArLS representing a range of countries and institutions. Strategically marketing the LIC brand, improving the LIC program profile within institutions by bridging logistics, and the need to scaffold the transition to the rural LIC learning environment emerged as key themes for success. The attendees highlighted their experiences of using peer groups, early exposure to rural LIC sites, and student allocation strategies for promotion. Unique learning styles adopted in LIC models, student anxiety and the importance of fostering supportive relationships with stakeholders to support students in their transition to the LIC environment were discussed.

**Discussion:**

This PeArLS highlighted successful systems and processes implemented in rural settings across different countries to recruit and manage the transition of medical students to rural LIC. The process proved to be a quick and efficient way to elicit rich information and may be of benefit to educationists seeking to establish similar programs or improve existing rural LIC.

## Background

Longitudinal integrated curricula have been increasingly used in medical schools around the world as the focus of medical education shifts towards an outcome-oriented model [1]. Longitudinal Integrated Clerkships (LIC) [2] are a subset of longitudinal integrated curricula that facilitate longitudinal and continual integration of education in clinical settings [3]. While traditional medical school programs involve a sequence of short clerkships, LIC involve participation in comprehensive care of patients over time fostering continued learning relationships between students, patients and clinicians to address the majority of an academic year’s core clinical competencies through these experiences [1, 4]. LIC placed in rural community settings have positively impacted the provision of rural health care services and promote rural career choices [5–9]. Through immersive longitudinal placement, students develop a strong sense of belonging by fostering connectivity through repeated interactions with the community [10]. This connectedness is not only a powerful tool for student agency and learning within LIC [11, 12], but can help to enrich the student experience which in turn can be vital in addressing workforce shortages in rural and remote communities.

The recruitment and engagement of students in rural medical training programs have significant challenges [13]. An annual Australian survey of medical students attending a rural clinical school indicated ~ 65% of students preferentially chose this placement with the remainder being filled by students with a lower preference [14]. Some of these students may not frequently exhibit traits associated with successful learning in a rural placement (high self-directedness, persistence and cooperativeness) [15] and may need additional support to transition to the unique learning environment in rural settings [16, 17].

The objectives of this study were to identify effective methods of recruiting medical students into rural LIC and supporting their transition to the LIC learning environment. The findings will be useful to medical schools who have implemented or are considering developing or implementing rural LIC.

Research question:

- What approaches can be utilized when recruiting and supporting the transition of medical students into rural LIC?

## Methods

We used a constructivist paradigm to frame the research question. We adopted a qualitative approach to explore the perspectives of those who have experience with LIC. The research team has a wealth of experience in designing and running rural LIC. The project was approved by Griffith University Human Research Ethics Committee (2021/676).

### Study setting and population

The study took place during the annual conference of the Consortium of Longitudinal Integrated Clerkships, a virtual event held from 10–13 October 2021, hosted by Stellenbosch University, South Africa. The Consortium is a world-wide network of university-based medical educationalists engaged in development or delivery of LICs for medical students [18]. Participants consisted of conference delegates attending the Personally Arranged Learning Session (PeArLS) themed ‘Secrets to success’.

### Personally Arranged Learning Sessions (PeArLS)

PeArLS allow presenters to introduce a challenge or a dilemma to a group of participants and benefit from collective problem-solving from the group [19]. Sessions are structured with five minutes for the presenter to introduce the topic and pose three to four questions to be discussed, 35 min of facilitated discussion, and five minutes for wrap-up. Facilitators are external to the presenting team and are nominated by the conference organizing committee.

The aim of this PeArLs was to continue to build a Community of Practice to achieve better outcomes for LIC. Communities of Practice have been extensively used in the field of education and their ability to develop the knowledge and skills of rural and remote educators has been widely acknowledged [20, 21]. The four questions posed were:

1. What strategies do you follow to attract sufficient students to fill rural LIC placements?
2. What tips would you suggest for enhancing the profile of a LIC?
3. How do you support students to preference and transition to an LIC model of learning and teaching?
4. What can we do better to support students integrate both to the rural community and community of practice?

### Data collection and analysis

Participants provided informed consent to participate in the study and the session was recorded by the facilitator with participants’ consent. Participants were encouraged to contact the facilitator within two weeks of the conference if they wished to provide any additional information regarding their LIC experiences.

The session recordings were transcribed verbatim using Sonix® transcription software and checked for data accuracy. Once transcribed and checked for accuracy, the transcripts were made pseudo-anonymous by substitution of any identifiers.

Following this process, the session transcripts were analysed by two members of the research team (JP, SW) using methods of constant comparative analysis. This is a process of thematic, open, axial, and selective coding that is used to extract themes in qualitative research [22]. Memos were kept to provide evidence of the analytic process and the decisions made to develop concepts and comparisons by the two researchers. Themes and patterns that were salient in the session were isolated by SW and then validated by JP and RH as to their relevance and applicability. Reliability and consistency were maintained through iterative and constant comparison approaches to the dataset (transcripts) [23]. NVivo® qualitative data management software was used for ease of data management during the analysis.

## Results

**Fig. 1:**
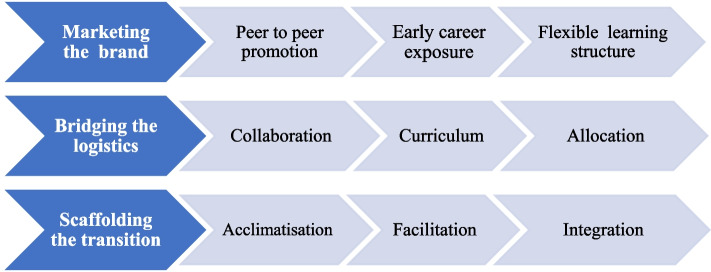
Themes and sub-themes

Thirteen participants attended the session and were representative of the following countries: Australia, South Africa, United Kingdom, United States and Taiwan. Of them, only six actively engaged in the discussion. Participants represented medical schools both in developed and developing countries and consisted of rural clinical school directors, program coordinators and a recent graduate of an LIC program. Themes and subthemes generated from the information shared by the participants are given in Fig. 1.

### Marketing the brand

Participants outlined the various methods they used to facilitate the recruitment of medical students to rural LIC. The importance of promotion using peer groups, early exposure to rural LIC sites, and clear communication of the learning styles adopted in rural LIC compared to traditional block rotation programs were highlighted.

#### Peer-to-peer promotion

Peer-to-peer promotion was described as one of the most effective methods of recruiting students to rural LIC. Several participants indicated that they utilised current LIC students and alumni as resources to promote rural LIC program to prospective students.

I can talk for an hour about the benefits of a LIC to the class, but if somebody that's been through the LIC, a student or a graduate, talks, they listen. (P1)

for me personally… having a session during my first year where I went out to the campus and heard from three or four or five students about what it was like and what the process was like and what the benefits and what the challenges were really stimulated me in my journey towards the LIC and gave me the confidence to continue through my LIC. (P2)

Students talk to students… students are our greatest advocate when it comes to the program of learning. (P3)

#### Early career exposure

Participants reported that early exposure of students to rural LIC sites and site-based excursions or activities were effective recruitment strategies. Expeditious showcasing of the rural LIC demystified the uncertainty often attributed to LIC and rural programs.

We found things that stimulate students to get an interest in our LIC is having excursions out to some of these sites relatively early on in the curriculum, taking students for site visits or having some activities happening there. (P1)

The role of early curriculum access to short term electives at rural LIC sites was highlighted as a mechanism for building awareness of rural LIC and assisting students to transition to LIC. Undertaking electives at LIC sites helped students to get a feel for the context and type of learning provided at these sites and facilitated a greater comprehension of the expectations and realities of participating in a rural LIC.

One of the things that we found that students did of their own accord was actually to do electives at sites where we have our LIC. And we actually tried formally at one point to do what we call a pre-LIC, to actually let students formally come and do an elective… we've seen a difference for those that came in sort of feeling already comfortable in the context and knowing what to expect when they started their LIC. (P4)

#### Flexible learning structure

The flexibility of the learning structure and greater levels of hands-on experience were recognized as unique features of rural LIC. The strong andragogical underpinnings of LIC had to be brought to the notice of the student cohort.

I think it's important to be kind of upfront and honest with students that the experience on a LIC or rural LIC might be different and won't give you the same experiences, but that the alternatives that you get could be even better for you and your development as a young healthcare worker. So really, marketing the things that a LIC can do - that no other method of learning can. So, focusing on things like workplace readiness and practical exposure: that normal academic ward rounds won’t be able to teach. (P2)

It was suggested that students who prefer flexible learning styles with greater hands-on experience may be more likely to choose and succeed in rural LIC.

Thinking about the students who choose LIC, generally, for us, it's those who want that learning style… it's more hands on and there’s greater participation over time and more flexible learning structure. And that's often what they're saying. That's why they're choosing it and they're also prepared to go rural. But those are often the things, perhaps to emphasize that are really positive. (P5)

The participants recognized that LIC involve a learning process that is different to more conventional processes which students are generally used to. One of the strategies that was discussed to support students in the transition to LIC was to educate students about the involved learning philosophies or processes.

I posted a picture in the chat that's from a 2021 article [24], and it actually graphically describes a J curve of learning, which David Hirsh [co-founder of Harvard Medical School Cambridge Integrated Clerkship [25]] often talks about. And I find that very useful to explain the learning within a LIC. So, when students come, we explain that there’ll be that feeling of loss, that you're not getting that specialist input and you're not having this advanced rare disease knowledge, but you, you're learning how to learn. (P1)

### Bridging the logistics

The participants shared logistical strategies that had proved successful in improving the profile of rural LIC within institutions. The strategies aimed at improving the LIC profile were believed to have a direct impact on student recruitment to rural LIC. These included collaboration with other organizations, improving integration between the rural LIC and the broader curriculum, and student allocation strategies.

#### Collaboration

The profile of rural LIC was recognized by participants as important to the success of the program, both in terms of recruiting students and in receiving support from participating institutions and communities. The participants highlighted the opportunities for, and benefits of collaboration with other organizations and associations to assist in enhancing the profile of rural LIC.

What rural does well and is, you know, probably our great strength here… is that we collaborate well. … So, we've got an opportunity to build on, you know, what are the collective value or collective things about rural. (P3)

Participants also shared experiences of how enhancing collaboration between students and local governments could be used to raise the profile of rural LIC in the rural community.

What we also found was that sometimes when we invited the local government leaders of the town, that students would actually connect with them with projects that they had envisaged but didn't have the capacity to do. (P6)

#### Curriculum

Opportunities for integration between rural LIC and the broader curriculum were discussed and were found to be of benefit for enhancing the profile of rural LIC. These included the incorporation of LIC principles in the curriculum and rural programs taking responsibility for components of assessment.

…when it came to a curriculum renewal process, many of our LIC principals are now incorporated in the renewed curriculum. And that's really strengthening the case for our LIC because we can tell students: Listen, this program is being incorporated into the new one, this is the way things are going. This is the future. So come. (P1)

When rural staff were responsible for components of assessments, participants felt that it had a positive impact on the student views of the rural program.

I've had experiences where rural programs have taken on responsibility for components of assessment, which has then influenced student's views about where, you know, the influence of assessment is, for example. (P3)

#### Allocation

The timing of and strategies for the allocation of students to rural LIC were discussed as a means of improving student uptake of rural LIC and assisting them in the transition.

Students come, do year one and two on campus… and really the natural, you know, the flow is then to stay… it’s the largest clinical school, that’s where all their peers are, that’s their social network, and so to do anything else is outside their comfort zone, basically. And we haven't had a lot of that pre-information or exposure to the site. So even more so, they're going somewhere unknown. And I think that's really the biggest battle. (P5)

Directly enrolling rural background students into a rural training stream, linked with a rural clinical school, was presented as a means of recruiting students at entry into medicine.

So, with our rural training stream next year, there's a few things we're putting together. One is recruiting rural background students from our actual footprint and then those students will… be matched to Rural Clinical Schools for years three and four. (P5)

### Scaffolding the transition

The importance of supporting students through the transition to rural LIC was recognized as a key aspect of the retention and success of students in these rural programs. Aspects that were highlighted by the participants included the importance of orientation and practical skill building, managing student anxiety relating to the transition, the utilization of support staff, and fostering supportive relationships with the medical and rural communities.

#### Acclimatization

Participants acknowledged that students face numerous difficulties when transitioning to rural LIC. The timing of allocation was recognized as important in assisting students to successfully transition to LIC and reduce any associated anxiety.

We’ve moved forward the clinical allocation as well so that they're allocated earlier so they haven't got their mind set. Because so often… when they get a rural clinical school and it wasn't their top preference, they're really thrown by it… So, if we allocate them earlier and it wasn't their preference, you know, they've got more time to adjust. (P5)

Participants also emphasized the importance of good orientation to LIC training sites.

Orientation is around explaining how the system works and what the pressures and things are so that they aren’t disorientated for as long, because you're always disorientated when you come into a new environment. (P3)

The idea of introducing some useful clinical skills to students early on so that they can actively participate as a member of the team was also presented as a strategy to assist in successful acclimatization.

… often students feel worried that they will be a burden, so sometimes stepping back and not having such an active role as an authentic member of the team can be out of concern that they don’t have anything valuable to give or that they’re going to be burdensome. So, teaching some things right up front will be of value and useful. (P3)

#### Facilitation

The role of mentors as advocates and enablers for students to help facilitate clinical opportunities was discussed.

We've actually employed a staff member or like an academic clinician in each of our major locations… Their main role is to enable the students in the clinical environment and to facilitate the clinical opportunities if they're not presenting well, so they become an advocate and enabler. (P3)

In addition, it was recognized that support staff are generally more readily available than clinical or academic staff, and that students may feel more comfortable in sharing challenges or difficulties with them.

One of the things that I've experienced was the value of support staff in marketing and being more readily available to the students... I think that is a hugely untapped resource at universities in particular. (P6)

#### Integration

The importance of assisting students to integrate into the medical workforce was highlighted by the participants.

…some of our rural sites have a very stable medical workforce. And so it's much easier to engage students into a group that's already quite stable and welcoming, particularly if there's a formal or informal leader of that group. But I think it's much more difficult, we've found in some of our sites that have a very locum dependent hospital environment because it's not a community of practice... (P3)

Integration into the rural community was identified as another important aspect of a successful rural LIC.

…one of the things that we did initially was when students went to a site, we would invite community leaders, community in all various aspects to an event to meet the students, and that kind of helped to a certain degree. (P6)

Some medical schools used more formal methods to integrate the community component to their programs.

We have a designated community representative at each of our sites… that person is nominated by the local community. (P5)

## Discussion

This PeArLS has resulted in a paradigm to guide recruitment and support the transition of students from metropolitan locations into rural longitudinal placements based on the combined experience of the participants.

Local and central leadership is vital in marketing rural LIC to the target audience [27]. The importance of champions for LIC programs, both among student peer groups and faculty leadership, has been previously highlighted [27–29]. Champions at both levels must develop and sustain relationships with key stakeholders and help facilitate rural LIC placements through early incorporation of rural sites and promotion of LIC programs. Early incorporation could be achieved through excursions, activities or electives on site with an aim to demystify and raise awareness of rural LIC placements. Peer-to-peer promotion and engagement through students was reported as the most efficient way to effectively raise awareness and recruit students. To strengthen this approach, champions may need to deliberately formalize these interactions into the program in a highly visible and accessible manner.

Bridging logistics in a rural LIC requires collaboration with relevant stakeholders, an autonomous yet flexible curriculum and considered student allocation. Collaboration with local and other partners was described as a key strength of rural LIC. Fostering collaborative relationships is mutually beneficial and enhances the sustainability of health services and medical schools [26]. A symbiotic curriculum where relationships are established and maintained between the key stakeholders in healthcare, is considered an ideal model for delivery of medical education [26]. As medical students are placed in the center of these fundamental symbiotic relationships, recruitment and support of students who acknowledge the unique learning environment of rural LIC is imperative to its success. The educational benefits achieved through delivering an LIC model could be used to drive positive change and curriculum renewal. This was seen to help promote the “best-practice” educational principles associated with LIC and hence a desire for students to participate in rural LIC. In addition, changes to standard curriculums may impact positively on the future integration of LIC as the educational principles and delivery of curriculum become more aligned. Achieving change requires establishment and fostering of relationships with clinicians, academics, students and rural communities [27]. The relationships built should be perpetual and achieve a shared understanding of LIC expectations and learning outcomes whilst recognizing the value of the curriculum in their different educational contexts [30]. The unique learning outcomes and expectations characteristic of LIC should also be clearly modeled, including benefits to students and stakeholders [27].

To further enhance recruitment strategy, known positive influences on workforce retention such as recruitment of students with a rural background should be given careful consideration [31]. The PeArLS’ participants recognized that commencing medical school in a larger clinical site negatively influenced transition to a rural location in the subsequent clinical years. Overcoming this could involve early career exposure across multiple year levels to build up exposure [12, 32], early allocation and peer-to-peer recruitment outlined previously. Other suggested solutions were engaging with rural background students, especially those from the LIC’s placement footprint and specific enrolment in a rural stream on admission. Therefore, strengthening rural connections and building on existing rural relationships may be a useful approach to optimize engagement in rural LICs.

During recruitment, attributes believed to be most suited for successful rural placements should be considered, with the goal of improving future rural workforce supply. Eley et al. [33] describe the “ideal” personality traits for medical students suited to rural practice as low harm avoidance, persistence, self-directedness, and cooperativeness. Aspects of rural LIC such as the flexible learning structure and hands-on experience may be more attractive to individuals with these personality traits. Emphasizing these aspects of rural LIC during recruitment may assist in recruiting students who are most suited to rural practiceWhile medical students who express an interest in rural placements are more likely to hold “ideal” personality traits, rural and metropolitan medical students exhibit similar trait profiles overall [33]. Caution must be taken if incorporating a concept of ideal characteristics as it may have exclusionary, harmful and discriminatory repercussions [34] on patients, the profession and the well-being of the student [35].

A strong support structure should be in place to facilitate the transition to rural LIC regardless of the personality traits or characteristics of the students enrolled. Transitioning to a rural LIC involves a sudden change to an unfamiliar educational environment, often without adequate signposting. This may be anxiety-provoking, lead to initial confusion or contribute to burnout in students. Whilst the process follows the J-curve [24], eventually resulting in gains in confidence and proficiency [36], contributors to the PeArLS recognized that the transition to a rural LIC requires implementing supportive structures and providing scaffolding. This vital role requires orientation to placement, practical skill building, managing student uncertainty, utilizing support staff and understanding the learning philosophies or processes involved in LIC programs [27]. Effective orientation to site including clear descriptions of the systems, pressures, and differences between rural LICs and metropolitan sites, was thought to reduce disorientation. This view is supported by Chou et al. [37], who found that clarification of roles and tasks, management of interpersonal challenges and addressing social and educational isolation helped to tackle disorientation. Development of clinical skills improved the students’ perceptions of their own authenticity as part of the clinical team. Early teaching of these skills prior to or at the commencement of clinical placements was suggested as the most successful approach.

Supportive relationships are needed at all levels of the symbiotic model [27]. This support can operate as a formal scaffolding for the student’s placement or as an informal confidante. The PeArLS participants emphasized the contributions made by on-site clinical and administrative staff in creating a supportive learning environment. The role of local champions is highlighted in previous work by Bartlett et al. [27]. A stable medical workforce was seen as a key element of a welcoming environment as well as continuity of formal and informal leadership on-site. Furthermore, integration into the community through consultation and engagement with community leaders and groups was noted as an important aspect of rural LICs. This approach has also been shown to deliver wider benefits to the community including workforce recruitment, retention, social benefits and local economic development [38].

Limitations to the study included the small participant sample of self-selecting delegates attending this session. The session was conducted live and subsequently crossed multiple time zones potentially impacting participation.

## Conclusions

This PeArLS aimed to develop a model for existing and future rural LICs to promote, recruit and support the transition of students. The session resulted in the collation of information from an audience with experience in running LIC programs to produce a paradigm which is supported by previous research in this area. Systems and processes that work in a variety of LIC models implemented in rural settings across different countries to recruit and manage the transition of medical students to rural LICs were highlighted. Further research evaluating the application of this paradigm will be required to determine its practicality and success in recruiting and transitioning medical students to rural LICs.

## Data Availability

The deidentified transcriptions are available from the corresponding author on reasonable request.

## References

[1] Worley P (2016). A typology of longitudinal integrated clerkships. Med Educ.

[2] Adams J (2020). Reflective writing as a window on medical students’ professional identity development in a longitudinal integrated clerkship. Teach Learn Med.

[3] Hense H (2021). Implementing longitudinal integrated curricula: systematic review of barriers and facilitators. Med Educ.

[4] Norris TE (2009). Longitudinal integrated clerkships for medical students: an innovation adopted by medical schools in Australia, Canada, South Africa, and the United States. Acad Med.

[5] Brown MEL, Anderson K, Finn GM (2019). A narrative literature review considering the development and implementation of longitudinal integrated clerkships, including a practical guide for application. J Med Educ Curric Dev.

[6] Somporn P, Ash J, Walters L (2018). Stakeholder views of rural community-based medical education: a narrative review of the international literature. Med Educ.

[7] Thistlethwaite JE (2013). A review of longitudinal community and hospital placements in medical education: BEME guide no. 26. Med Teach.

[8] O’Sullivan BG (2018). A review of characteristics and outcomes of Australia’s undergraduate medical education rural immersion programs. Hum Resour Health.

[9] Kitchener S (2015). Longlook: initial outcomes of a longitudinal integrated rural clinical placement program. Aust J Rural Health.

[10] Daly M (2013). Longitudinal integrated rural placements: a social learning systems perspective. Med Educ.

[11] Roberts C (2017). Social learning in a longitudinal integrated clinical placement. Adv Health Sci Educ Theory Pract.

[12] Carrigan B, et al. Connectivity is the key to longer rural placement: retaining students on rural longitudinal integrated clerkships. Med Teach. 2023:1–7. 10.1080/0142159X.2023.2243025 37557884

[13] Wolcott MD (2021). Using design thinking to explore rural experiential education barriers and opportunities. J Med Educ Curric Dev.

[14] Isaac V, et al. FRAME Survey Report October 2020. Federation of Rural Australian Medical Educators; 2020.

[15] Eley DS (2014). Toward a global understanding of students who participate in rural primary care longitudinal integrated clerkships: considering personality across 2 continents. J Rural Health.

[16] Eley DS, Young L, Przybeck TR (2009). Exploring the temperament and character traits of rural and urban doctors. J Rural Health.

[17] Eley DS (2017). Tolerance of ambiguity, perfectionism and resilience are associated with personality profiles of medical students oriented to rural practice. Med Teach.

[18] The Consortium of Longitudinal Integrated Clerkships. About. 2022. Available from: https://clicmeded.com/about/. Cited 2022 25 September.

[19] The Consortium of Longitudinal Integrated Clerkships. Core elements. 2022. Available from: https://clicmeded.com/. Cited 25 Sept 2022.

[20] Walton E (2019). What matters in learning communities for inclusive education: a cross-case analysis. Prof Dev Educ.

[21] Vescio V, Ross D, Adams A (2008). A review of research on the impact of professional learning communities on teaching practice and student learning. Teach Teach Educ.

[22] Grove RW (1988). An analysis of the constant comparative method. Int J Qual Stud Educ.

[23] Davidson A (2021). Validating an obstetrics and gynaecology longitudinal integrated clerkship curriculum at the University of Toronto: a four-year review. J Obstet Gynaecol Can.

[24] Brown MEL (2021). Not all who wander are lost: evaluation of the Hull York medical school longitudinal integrated clerkship. Educ Prim Care.

[25] Dutchen S. Academy Accolades, David Hirsh is inaugural Thibault Academy Associate Professor. 2018. Available from: https://hms.harvard.edu/news/academy-accolades. Cited 2022 Oct 25.

[26] Bartlett M (2020). The do’s, don’ts and don’t knows of establishing a sustainable longitudinal integrated clerkship. Perspect Med Educ.

[27] Bartlett M (2019). Dundee’s longitudinal integrated clerkship: drivers, implementation and early evaluation. Educ Prim Care.

[28] Ellaway R (2013). Twelve tips for designing and running longitudinal integrated clerkships. Med Teach.

[29] Prideaux D, Worley P, Bligh J (2007). Symbiosis: a new model for clinical education. Clin Teach.

[30] Schrewe B (2018). The contextual curriculum: learning in the matrix, learning from the matrix. Acad Med.

[31] Laven G, Wilkinson D (2003). Rural doctors and rural backgrounds: how strong is the evidence? A systematic review. Aust J Rural Health.

[32] Kerr A (2021). Early longitudinal community pharmacy placements: connection, integration and engagement. Res Social Adm Pharm.

[33] Eley DS (2019). The personalities of most medical students are suited to rural practice: implications for rural education program recruitment. Med Teach.

[34] Allitt M, Frampton S (2022). Beyond ‘born not made’: challenging character, emotions and professionalism in undergraduate medical education. Med Humanit.

[35] Powis D (2020). Why is it so hard to consider personal qualities when selecting medical students?. Med Teach.

[36] Dube TV (2015). Transition processes through a longitudinal integrated clerkship: a qualitative study of medical students’ experiences. Med Educ.

[37] Chou CL (2014). Workplace learning through peer groups in medical school clerkships. Med Educ Online.

[38] Couper I, Worley PS, Strasser R (2011). Rural longitudinal integrated clerkships: lessons from two programs on different continents. Rural Remote Health.

